# Editorial: Psychosocial risk factors in the development, maintenance and treatment outcome of eating disorders

**DOI:** 10.3389/fpsyg.2024.1486941

**Published:** 2024-09-12

**Authors:** Matteo Aloi, Marianna Rania, Gianluca Lo Coco, Antonino Carcione, Giovanni Castellini, Karin Waldherr, Cristina Segura-Garcia

**Affiliations:** ^1^Department of Clinical and Experimental Medicine, University of Messina, Messina, Italy; ^2^Outpatient Unit for Clinical Research and Treatment of Eating Disorders, University Hospital Renato Dulbecco, Catanzaro, Italy; ^3^Department of Psychology, Educational Science and Human Movement, University of Palermo, Palermo, Italy; ^4^Third Centre of Cognitive Psychotherapy, Italian School of Cognitive Psychotherapy (SICC), Rome, Italy; ^5^Department of Human Science, University “Guglielmo Marconi”, Rome, Italy; ^6^Psychiatry Unit, Department of Health Sciences, University of Florence, Florence, Italy; ^7^Ferdinand Porsche FernFH, Distance Learning University of Applied Sciences, Wiener Neustadt, Austria; ^8^Department of Medical and Surgical Sciences, University “Magna Graecia” of Catanzaro, Catanzaro, Italy

**Keywords:** eating disorders, risk factors, treatment, onset, maintenance, development

Eating disorders (EDs) are psychiatric conditions characterized by pathological eating behaviors and attitudes toward food and body image (American Psychiatric Association, 2013). While genetic and biological factors play a significant role in the development of EDs (Huckins et al., 2024), psychosocial risk factors are considered crucial to understanding their onset and persistence (Aloi et al., 2022). These factors include a wide range of influences, such as familial dynamics, societal pressures, traumatic experiences, and individual psychological characteristics.

The family environment, including parental attitudes toward weight and diet, critical or controlling parenting styles, and a history of EDs in the family, can significantly impact an individual's relationship with food and body image (Gorrell et al., 2022). Societal and cultural pressures, particularly the pervasive idealization of thinness and appearance promoted by media and social platforms, further exacerbate body dissatisfaction and unhealthy eating behaviors (Holland and Tiggemann, 2016; Saiphoo and Vahedi, 2019).

Traumatic experiences, including physical, emotional, and sexual abuse, are also strongly linked to the development of EDs (Convertino and Mendoza, 2023). These experiences can lead to the development of maladaptive coping mechanisms, such as disordered eating, to regain a sense of control or numb emotional pain. Additionally, individual psychological factors, such as low self-esteem, perfectionism, interpersonal problems and difficulties with emotional regulation, play a pivotal role in both the development and maintenance of EDs (Robinson et al., 2024; Robinson and Wade, 2021; Lo Coco et al., 2012).

Therefore, the aim of this Research Topic is to enhance our understanding of how individual psychosocial factors can impact EDs, often exacerbating the clinical course and leading to less effective treatments.

In terms of social pressures factors, Cerolini et al. explore how body shaming and internalized weight bias contribute to the development of EDs among adolescents. They emphasize the pervasive nature of weight stigma and how societal pressures about body image can lead to harmful psychological effects. Adolescents internalize these negative attitudes, which can significantly impact their self-esteem and body satisfaction. The study highlights the strong association between internalized weight bias and the onset of disordered eating behaviors, suggesting that addressing these psychosocial factors is crucial for effective prevention and intervention strategies. Moreover, the study underscores the need for comprehensive programs targeting weight stigma and promoting body positivity to mitigate the risk of developing EDs in this vulnerable population.

In line with this topic, an interesting study by Bogár et al. investigates the prevalence and impact of EDs and body image issues within the fashion industry. The study employs content analysis to examine how the intense environmental pressures faced by fashion models contribute to the development and exacerbation of these disorders. It highlights the role of factors like industry standards, media portrayal, and occupational stress in shaping the mental health of models. The findings suggest that the high demands and unrealistic beauty standards perpetuated by the fashion industry significantly increase the risk of EDs and body image disturbances among models. The manuscript calls for heightened awareness and intervention strategies to address these psychosocial risk factors, aiming to foster a healthier and more supportive environment within the fashion industry. The research accentuates the need for systemic changes to protect the wellbeing of individuals in this high-pressure profession.

Two additional studies delve into the significant topic of overvaluation of weight, shape, and physical appearance in EDs. In the first study, Escandón-Nagel et al. aim to understand how overvaluation of weight and shape differs between individuals with overweight with and without binge eating disorder (BED). Through the Repertory Grid Technique, the research seeks to identify how self-construction and cognitive structures are associated with overevaluation in obesity, both with or without BED. The findings indicate that individuals with BED exhibit significantly higher levels of weight and shape overvaluation compared to those without BED. This overvaluation is associated with greater ED psychopathology and poorer mental health outcomes. The study highlights the importance of addressing self-construction and cognitive structure in treatment approaches for BED, suggesting that an approach based on Kelly's Personal Construct Theory focusing on weight and shape concerns may be particularly beneficial for this population. This research contributes to the nuanced understanding of the psychological factors underlying BED and obesity.

In this context, the second study by Schönhals et al. evaluates the psychometric properties of the German versions of the Upward and Downward Physical Appearance Comparison Scales (UPACS and DACS). The research involved both a clinical sample and a control group, with a total of 2,114 participants. Structural equation modeling confirmed the one-factor structure of the DACS but not the UPACS. Both scales demonstrated good internal consistency and test-retest reliability. They also correlated as expected with related constructs such as appearance comparisons, eating disorder pathology, and self-esteem, indicating acceptable construct validity. However, some limitations of the UPACS were noted for women with EDs. Overall, this study suggests that both the UPACS and DACS are psychometrically suitable tools for assessing upward and downward physical appearance comparisons in both research and clinical settings, across genders and eating disorder statuses.

Regarding genetic risk factors, the systematic review by Almaghrbi and Bawadi explores the intricate relationship between gene polymorphisms and psychological/neurobiological factors in individuals with anorexia nervosa (AN). Drawing from 11 selected studies, the review highlights significant findings across various genetic markers and their impact on AN. Key insights include the consistent association of the 5-HTTLPR polymorphism with altered connectivity in brain networks, impaired inhibitory control, and heightened susceptibility to AN. Additionally, genes within the dopaminergic system (e.g., COMT, DRD2, DRD3, DAT1) influence reward processing, motivation, and decision-making processes implicated in AN pathology. The Val66Met polymorphism in the BDNF gene influences personality traits, eating behaviors, and emotional responses across different populations. Other genes, such as OXTR, TFAP2B, and KCTD15 are associated with social cognition, emotional processing, and body image concerns in AN. These findings underscore the genetic complexity underlying AN and advocate for further research to enhance therapeutic strategies tailored to genetic predispositions.

Furthermore, the systematic review of Di Luzio et al. explores the similarities and differences in clinical, genetic, and neurobiological aspects between Obsessive-Compulsive Disorder (OCD) and EDs, particularly during childhood and adolescence, which are crucial phases of neurodevelopment. Through a systematic review of 10 selected articles, OCD symptoms resulted more prevalent in individuals with EDs characterized by binge/purge behaviors compared to restrictive profiles. Both disorders exhibit obsessive-compulsive symptomatology, suggesting shared neurobiological alterations, particularly in the anterior cingulate cortex, which affects cognitive flexibility. Genetic overlaps between OCD and EDs were also identified. The study underscores the importance of integrating psychopathological and neurobiological perspectives for accurate diagnosis and tailored treatment strategies, potentially outperforming the outcome.

In the context of family environment, an interesting study by Bevione et al. investigates the impact of parental educational level (PEL) on EDs, focusing on treatment compliance and outcomes. The study highlights that patients with higher PEL, whether from mothers, fathers, or both, exhibit higher depressive symptoms but also lower levels of parental criticism. These patients tend to be younger, have an earlier onset of EDs, and shorter illness durations. Those with fathers or both parents with higher PEL are more likely to have AN, longer hospital stays, and higher personal standards, while those with mothers with higher PEL show lower rates of substance andalcohol addiction. The study also reveals that personal standards mediate the relationship between higher PEL and lower dietary restraint. Overall, PEL emerges as a multifaceted risk factor influencing various aspects of EDs, suggesting nuanced pathways for intervention and support in clinical settings.

A stimulating study examines the role of perceived stress in relation to binge eating behavior in Chinese university students, focusing on the roles of life history strategy and distress tolerance. Li et al. found that perceived stress influences binge-eating behavior through its impact on life history strategy, suggesting a mediation effect. Additionally, distress tolerance was found to moderate both the direct relationship between perceived stress and binge-eating behavior, as well as the indirect relationship mediated by life history strategy. These findings imply that enhancing distress tolerance skills and implementing interventions based on life history theory could be effective to mitigate binge-eating triggered by perceived stress among university students in China. This study contributes to understanding the psychological mechanisms underlying binge eating in the context of stress, offering potential avenues for targeted interventions.

An important contribution regarding childhood adversities is the systematic review by Johnsen et al.. This systematic review synthesizes evidence on the prevalence of non-interpersonal traumatic events (NTE) in individuals with EDs. Conducted following the PRISMA guidelines, this review included 16 studies that identify five main types of NTE: accidents, illness, injury, natural disasters, and war. The findings suggest that illness and injury were more prevalent among patients with EDs compared to controls, particularly in those with the binge/purge subtype of AN. However, other types of NTE did not consistently show higher prevalence among patients with EDs. The review highlights a significant gap in research regarding the timing of trauma relative to EDs onset, which could influence associations. Overall, it emphasizes the need for more comprehensive studies to further understand the relationship between NTE and EDs, suggesting potential implications for treatment and intervention strategies.

Interestingly, two studies used the latent profile analysis (LPA) approach. LPA is a statistical method that identifies unobserved subgroups, or latent classes, within a heterogeneous population based on patterns of responses across multiple variables. Unlike traditional clustering methods, LPA assumes that individuals within each latent class share similar response patterns on several variables, but these classes are not directly observed or measured. By identifying these latent classes, LPA helps researchers understand underlying structures or typologies that exist within their data, revealing meaningful subgroups that may have distinct characteristics or behaviors. In the first study, Soodla et al. investigate the robustness of personality-based subtypes within patients with EDs through LPA, exploring various methodological approaches. Analyzing data from 221 individuals with EDs, the study conducted four LPAs, manipulating constraints on variances and covariances, and integrating state ED symptom measures as indicators. Results identified a stable four-profile model primarily defined by variations in impulsivity and perfectionism traits. Profiles with the most and least disturbances, both state and trait-related, were consistently replicated across different analytic conditions. Including ED symptoms enhanced subtype differentiation and alignment across profiles. In the second study, Aloi et al. examine metacognitive profiles among 395 individuals diagnosed with various EDs using LPA. The research identified three distinct profiles: high functioning (HF), intermediate functioning (IF), and low functioning (LF), characterized by different levels of metacognitive impairments. Participants in the IF group tended to be older with higher BMI compared to those in the HF and LF groups. Individuals with bulimia nervosa (BN) predominantly fell into the HF and LF profiles, while those with BED were mainly categorized in the IF profile. The LF group exhibited significant psychological distress, including high levels of depression, emotional dysregulation, childhood adversity, and personality dysfunction. Multinomial logistic regression highlighted associations between metacognitive profiles and factors such as emotional abuse, emotional dysregulation, and detachment. These findings underscore the potential for metacognitive interpersonal therapy, a treatment focused on reducing impairments in metacognitive abilities, as a targeted intervention for EDs based on distinct metacognitive profiles identified in this study.

Finally, regarding the outcome variables involved in EDs treatment, the study of Muzi et al. explores the impact of Ryff's model of psychological wellbeing (PWB) and the early therapeutic alliance on treatment outcomes for 165 female-assigned patients with EDs in a residential program. The authors found that dimensions of PWB such as autonomy, positive relationships, and self-acceptance were associated with clinically significant and reliable symptom reduction. Additionally, the early therapeutic alliance plays a crucial role, directly influencing treatment outcomes and mediating the relationship between PWB dimensions and overall symptomatic improvement. These findings highlight the importance of both individual psychological strengths and the therapeutic relationship in EDs treatment. Authors suggest that understanding and fostering PWB dimensions and early alliance development could enhance therapeutic effectiveness, potentially aiding in predicting and addressing challenges in the therapeutic process to optimize patient outcomes.

The manuscripts included in this Research Topic represent a step forward in the comprehensive description and explanation of psychosocial risk factors in EDs, providing practical and useful information for their assessment and application in different treatment options and, most useful and desirable, in prevention. Identifying these risk factors remains a major challenge for health and mental health professionals.

Overall, this Research Topic aims to contribute to the advancement of the field by providing valuable insights to help practitioners and researchers understand the complexity of psychosocial risk factors in EDs. It is hoped that this Research Topic of studies will lead to improved prevention and treatment strategies, benefiting all those involved in the more effective management of EDs.

## References

[B1] Aloi M. Lo Coco G. Carcione A. Nicolò G. Tasca G. A. Segura-Garcia C.. (2022). Editorial: Psychosocial risk factors in the development and maintenance of eating disorders. Front Psychol. 13:964626. 10.3389/fpsyg.2022.964626 35911003 PMC9328156

[B2] American Psychiatric Association (2013). Diagnostic and Statistical Manual of Mental Disorders, 5th Edn. Arlington, VA: American Psychiatric Publishing. 10.1176/appi.books.9780890425596

[B3] Convertino A. D. Mendoza R. R. (2023). Posttraumatic stress disorder, traumatic events, and longitudinal eating disorder treatment outcomes: a systematic review. Int. J. Eat Disord. 56, 1055–1074. 10.1002/eat.23933 36916450 PMC10247514

[B4] Gorrell S. Byrne C. E. Trojanowski P. J. Fischer S. Le Grange D. A. (2022). scoping review of non-specific predictors, moderators, and mediators of family-based treatment for adolescent anorexia and bulimia nervosa: a summary of the current research findings. Eat Weight Disord. 27, 1971–1990. 10.1007/s40519-022-01367-w 35092554 PMC9872820

[B5] Holland G. Tiggemann M. A. (2016). systematic review of the impact of the use of social networking sites on body image and disordered eating outcomes. Body Image 17, 100–110. 10.1016/j.bodyim.2016.02.008 26995158

[B6] Huckins L. M. Brennand K. Bulik C. M. (2024). Dissecting the biology of feeding and eating disorders. Trends Mol. Med. 30, 380–391. 10.1016/j.molmed.2024.01.009 38431502

[B7] Lo Coco G. Gullo S. Scrima F. Bruno V. (2012). Obesity and interpersonal problems: an analysis with the interpersonal circumplex. Clin. Psychol. Psychother. 19, 390–398. 10.1002/cpp.753 21538669

[B8] Robinson K. Wade T. D. (2021). Perfectionism interventions targeting disordered eating: a systematic review and meta-analysis. Int. J. Eat Disord. 54, 473–487. 10.1002/eat.23483 33594679

[B9] Robinson L. Flynn M. Cooper M. (2024). Individual differences in motivation to change in individuals with eating disorders: a systematic review. Int. J. Eat Disord. 57, 1069–1087. 10.1002/eat.24178 38436481

[B10] Saiphoo A. N. Vahedi Z. (2019). A meta-analytic review of the relationship between social media use and body image disturbance. Comput. Hum. Behav. 101, 259–275. 10.1016/j.chb.2019.07.028

